# A prospective observational study to explore the correlation of peripheral arterial pulse/resistance index, organ function, and inflammation in patients with septic shock

**DOI:** 10.1097/MD.0000000000020235

**Published:** 2020-05-15

**Authors:** Lirong Hu, Bo Leng, Changxue Wu, Yuting Xue

**Affiliations:** a ICU, Affiliated Hospital of Southwest Medical University; b Department of General Surgery, Affiliated Hospital of Traditional Chinese Medicine, Southwest Medical University; c Department of Critical Care, Luzhou Maternal and Child Health Hospital, Luzhou, Sichuan, China.

**Keywords:** blood vessel function, blood vessel pulsation index, fluid resuscitation, inflammation, resistance index, septic shock

## Abstract

**Introduction:**

The evaluation of the functional status of blood vessels, especially the arterial system, plays a very important role in the judgment of the condition of septic shock patients and the guidance of resuscitation programs and the judgment of the therapeutic effect. We aimed to design an observational study protocol to explore the correlation of peripheral arterial pulse/resistance index, organ function and inflammation in patients with septic shock.

**Methods and analysis:**

A total of 60 patients with septic shock in the Affiliated Hospital of Southwest Medical University from June 2020 to September 2020 and 20 healthy volunteers will be enrolled. Total of 60 patients with septic shock will be randomly divided into 20 groups by lot method. Group 1: fluid resuscitation; Group 2: fluid resuscitation + norepinephrine; Group 3: fluid resuscitation + norepinephrine + ulinastatin; Group 4: healthy control group. Fluid resuscitation is an early goal-directed fluid resuscitation in which norepinephrine is adjusted by a senior intensive care unit specialist for clinical presentation and ulinastatin is pumped at 20,000 U/h. Index including vascular ultrasound, inflammatory factors, organ function will be collected and analyzed.

**Discussion:**

Existing studies on septic shock focus on hemodynamics of the heart, brain, and kidney, while the differences in blood flow between peripheral blood vessels and protective renal vessels may be consistent, and imaging analysis is still lacking. This study protocol aims to explore the correlation of peripheral arterial pulsation index/resistance index, organ function, and inflammation in patients with septic shock.

**Trial registration:**

Chinese Clinical trial registry: ChiCTR2000031565

## 1 Introduction

Septic shock refers to septic syndrome with shock caused by microorganisms and their toxins. Septic shock usually results to damage of various organs, thus affecting the perfusion, cause tissue ischemia hypoxia, metabolic disorders, dysfunction, and even multiple organ failure. Septic shock is one of the most common causes of death in critically ill patients, with a mortality rate of up to 50%.^[1,2]^ In patients with septic shock, high doses of dopamine and norepinephrine still make it difficult to reverse hypotension. As the dose of catechol increases, powerful vasospasm leads to decreased perfusion of vital organs, increasing the incidence of cardiovascular adverse events.^[3]^ During the resuscitation of septic shock, it is necessary to pay attention to the matching of cardiac function, blood volume, ventricular ejection, and vascular tone.

The evaluation of the functional status of blood vessels, especially the arterial system, plays a very important role in the judgment of the condition of septic shock patients and the guidance of resuscitation programs and the judgment of the therapeutic effect. Blood vessel pulsation index (PI) and resistance index (RI) are commonly used method for the evaluation of blood vessel function, which reflect vascular bed resistance. RI is the measurement of systolic peak velocity (PSV) and end-diastolic blood velocity (EDV) through the arterial blood flow waveform. Calculation formula: RI = (PSV − EDV)/PSV; PI = (PSV − EDV)/mean blood velocity. These parameters can reflect the compliance and elastic resistance of a section of the artery. At present, researches on vascular PI and RI mainly focus on cerebral arteries, renal arteries, and coronary arteries, which are used to evaluate the effects of various chronic systemic diseases, including trauma, surgical intervention.^[4,5,6]^ However, the correlation of peripheral arterial PI/RI, organ function and inflammation in patients with septic shock need to be further investigated.

In this study, patients with septic shock will be selected, and the pulse index and resistance index of femoral artery, brachial artery and internal carotid artery will be analyzed by color vascular ultrasound Doppler technique, and the differences between peripheral artery and heart, brain, and kidney artery, as well as the correlation with the level of blood inflammatory factors will be analyzed. At the same time, different fluid resuscitation and vasoconstrictor drug regimens will be set up to compare the difference and treatment effect of peripheral arterial pulse index and resistance index in patients with different treatment regimens, so as to analyze the guiding significance of peripheral arterial pulse index and resistance index to treatment. Research results will be in the existing condition in patients with septic shock judgment on the basis of the traditional methods, add blood vessel function to judge the important indicator, more systematic comprehensive analysis of the circulation situation, guide the liquid recovery and drug use, is expected to be especially severe medical physicians for clinicians improve new means of increasing the success rate of patients with septic shock.

## 2 Methods and analysis

### 2.1 Main aims

Aim 1: To analyze the changes of peripheral arterial pulse index and resistance index in septic shock patients with color Doppler technique.

Aim 2: To explore the relationship between peripheral arterial pulse index and resistance index and inflammatory factor level in septic shock patients.

Aim 3: To perform the Correlation analysis of peripheral artery and cardiac, cerebral and renal blood flow and function in patients with septic shock.

Aim 4: To analyze the effects of fluid resuscitation and vasoactive drugs on peripheral blood pulse index and resistance index in septic shock patients.

### 2.2 Study registration

The protocol scheme matches PRISMA's reporting standards. This study protocol has been registered on the Chinese Clinical trial registry (http://www.chictr.org.cn/index.aspx) with a unique ID of ChiCTR2000031565 on April 4, 2020.

### 2.3 Participants

Sixty patients with septic shock (diagnosis according to the 2014 guidelines for septic shock) and 20 adult healthy volunteers will be enrolled in this study.

#### 2.3.1 Inclusion criteria

(1) Patients have clear symptoms of infection;
(2) Patients show systemic inflammatory response;
(3) The systolic blood pressure is lower than 90 mm hg, or the original base drops by 40 mm hg, which cannot be recovered after 1 hour after fluid resuscitation or requires vasoactive drugs to maintain;
(4) Accompanied by organ tissue hypoperfusion, such as urine volume less than 30 mL/h, or with acute consciousness disorders; Blood cultures may contain disease-causing microorganisms.

#### 2.3.2 Exclusion criteria

(1) The patient already has severe arrhythmia such as ventricular fibrillation;
(2) The patient has been diagnosed as brain dead;
(3) The patient has developed severe multiple organ failure;
(4) The patient had peripheral arterial lesions such as thromboangiitis obliterans.

### 2.4 Recruitment

Sixty patients with septic shock in the intensive care unit of Affiliated Hospital of Southwest Medical University from June 2020 to September 2020 and 20 healthy volunteers will be recruited. Written consent will be obtained from all participates, which confirms the participants’ voluntarism.

### 2.5 Grouping

A total of 60 patients with septic shock will be randomly divided into 20 groups by lot method. Group 1: fluid resuscitation; Group 2: fluid resuscitation + norepinephrine; Group 3: fluid resuscitation + norepinephrine + ulinastatin; Group 4: healthy control group. Fluid resuscitation is an early goal-directed fluid resuscitation in which norepinephrine is adjusted by a senior intensive care unit specialist for clinical presentation and ulinastatin is pumped at 20,000 U/h.

### 2.6 Data collection

Detection time point: before recovery, 6 hours, 24 hours recovery.

(1) Vascular ultrasound
  GE E9 color Doppler ultrasound diagnostic instrument will be used, and the vascular probe frequency will be 3.5 hz. In accordance with the routine requirements of the ultrasound department, the patient will be positioned to expose bilateral internal carotid artery, brachial artery, and femoral artery in turn. The vessel diameter will be measured by 2-dimensional figure, the blood flow velocity will be measured by color Doppler, and the blood flow waveform, pulse index, and resistance index will be analyzed by spectral Doppler. By the same method, the frequency and speed of the probe will be adjusted to measure the intracranial artery, coronary artery, and renal artery. Cardiac function will be measured routinely with a heart probe.
(2) Inflammatory factors
  Peripheral venous blood will be extracted and enzyme-linked immunosorbent assay will be used to analyze the levels of peripheral venous blood inflammatory factors, such as interleukin-2, 6, 8, 10, tumor necrosis factor-, cell adhesion molecule ICAM-1, endothelin ET, signal molecule NF- p in patients with septic shock.
(3) Organ function: according to clinical routine data collection and test results
  Cardiac function: heart rate, blood pressure, peripheral circulation, ultrasound, vasoactive drugs; renal function: urine volume, renal function test; brain function: consciousness, pupil, GCS score; Lung function: blood gas, ventilator use.

### 2.7 Statistical plan

SPSS 20.0 software will be used for data statistics and analysis by professional statisticians.

### 2.8 Ethics and dissemination

The study protocol was approved by the Affiliated Hospital of Southwest Medical University. The approved protocol was registered. Written informed consent will be obtained from all patients and volunteers. The privacy of all participants will be protected. The study will be performed in accordance with the standards of the International Committee on Harmonization on Good Clinical Practice and the revised version of the Declaration of Helsinki principles.

## 3 Discussion

The pathophysiological feature of septic shock is the cascade inflammatory response, which leads to circulatory dysfunction, resulting in the inability of the body's oxygen supply and the deterioration of tissue perfusion, thus in turn aggravating the body's inflammatory response. Due to the endogenous cytokines and inflammatory mediators, microcirculation network abnormal vascular tension forms a unique phenomenon named “vascular waterfall.”^[7,8]^ Vascular waterfall induced shock and high dynamic state and peripheral vascular paralysis coexist causes serious organ hypoperfusion and hypoxia. Therefore, improving organ perfusion, reverse hypoxia is the key to successful resuscitation.

Due to the high threshold of ultrasound detection technology for coronary arteries, renal arteries, intracranial arteries, and other vessels, it is not appropriate for general clinicians, especially for critical medical physicians, to quickly evaluate the vascular functional status beside the bed and guide the resuscitation treatment of septic shock. Based on these questions, this study protocol hypothesizes that peripheral arteries such as the femoral artery, brachial artery, and carotid artery, which were more easily detected, will be selected to replace the cardiac, cerebral, and renal vessels to help clinicians determine the functional status of vessels in the case of septic shock. In the case of septic shock, patients will show differences in blood flow distribution. Existing studies on septic shock focus on hemodynamics of the heart, brain, and kidney, while the differences in blood flow between peripheral blood vessels and protective renal vessels may be consistent, and imaging analysis is still lacking. This study protocol aims to explore the correlation of peripheral arterial PI/RI, organ function and inflammation in patients with septic shock.

## Author contributions

Changxue Wu conceived the idea and provided the fund for this study; Bo Leng provided statistical plan; Lirong Hu drafted the protocol. Yuting Xue reviewed the protocol and provided critical feedback. All authors approved the article to be published in Medicine.
